# Genome sequence of a novel *Tetraparvovirus* identified in a *Rhinophylla pumilio* bat from the Amazon region

**DOI:** 10.1128/mra.00593-25

**Published:** 2025-07-31

**Authors:** Leonardo H. Almeida Hernández, Thito Y. Bezerra da Paz, Sandro Patroca da Silva, Fábio Silva da Silva, Bruno C. Veloso de Barros, Lívia M. Neves Casseb, Pedro F. da Costa Vasconcelos, Ana C. Ribeiro Cruz

**Affiliations:** 1 Graduate Program in Virology, Evandro Chagas Institute, Health and Environment Surveillance Secretariat, Ministry of Health 89124, Ananindeua, Brazil; 2 Department of Arbovirology and Hemorrhagic Fevers, Evandro Chagas Institute, Health and Environment Surveillance Secretariat, Ministry of Health 89124, Ananindeua, Brazil; 3 Graduate Program in Parasite Biology in the Amazon Region, Pará State University, Belém, Brazil; 4 Faculdade Vale dos Carajás 89124, Parauapebas, Brazil; DOE Joint Genome Institute, Berkeley, California, USA

**Keywords:** parvovirus, bats, metagenomics

## Abstract

A new *Tetraparvovirus* was identified in a pooled tissue sample of a *Rhinophylla pumilio* bat from Santa Bárbara, Pará State, Brazil. This is the second *Tetraparvovirus* described in bats and has higher nucleotide and amino acid identity with a *Tetraparvovirus* from opossum, also described in Brazil.

## ANNOUNCEMENT

*Tetraparvoviruses*, family Parvoviridae, are linear single-stranded DNA viruses of ∼5 kb in length. They have two open reading frames: one for the NS1 replicase gene and another for the VP capsid gene (1). For *Tetraparvoviruses*, the NS1 amino acid identity is considered for species demarcation based on a >85% threshold (https://ictv.global/report/chapter/parvoviridae/parvoviridae/tetraparvovirus). As of February 2025, there are eight species classified in this genus, which have been identified in primates, ungulates, or bats. The one from bats, *Tetraparvovirus chiropteran1*, was identified in some *Eidolom helvum* from Ghana and has a genome of 5,065 nt. According to Canuti et al., spleen and kidney were identified as the potential replication sites of this virus (2). On 07 April 2015, tissue samples were collected from a healthy adult male *Rhinophylla pumilio* bat from Santa Bárbara in Pará State, Brazil (1.208333°S 48.270833°W). Fragments of its spleen, lungs, heart, and lymph nodes tissues were pooled prior to processing. This project was authorized by the Ethics Committee on Animal Use of the Evandro Chagas Institute under certificates 21/2014 and 10/2024.

RNA extraction was performed using the PureLink RNA Mini Kit (Invitrogen) associated with TRIzol Reagent (Invitrogen). The first and second strands of cDNA were synthesized via the SuperScript IV VILO MasterMix (Invitrogen) and the Second Strand cDNA Synthesis Kit (Invitrogen), respectively. A cDNA library was prepared with the Nextera XT DNA library preparation kit and sequenced on an Illumina NextSeq 500 platform with 150 bp paired-end reads. Metagenomic data analysis consisted of raw data quality control and cleaning by Fastp v0.23.4 (3), rRNA removal using SortMeRNA v.2.1 (4), *de novo* assembly via MEGAHIT v1.2.9 (k-mers 21, 31, 41, 51, 61, 71, 81, 91, and 99) (5), and alignment against the nr protein database by DIAMOND (6). Contig inspection, map-to-reference alignment (Geneious Mapper), and multiple sequence alignment (Clustal Omega v1.2.3) were performed using Geneious Prime 2024.0 (7). Phylogenetic analysis was performed with IQ-TREE v2.2.5 (8), using the maximum likelihood method and 1,000 bootstrap iterations. Default parameters were used except where otherwise noted.

**Fig 1:**
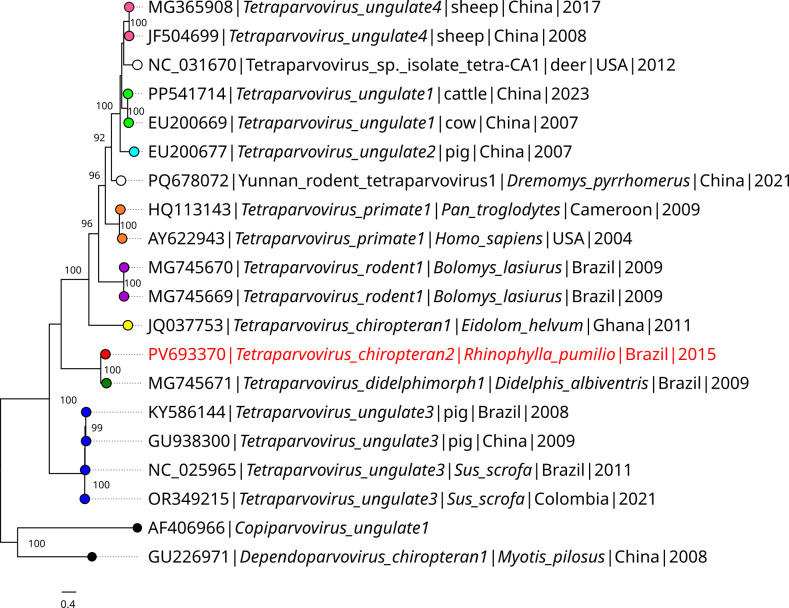
Maximum likelihood phylogenetic tree based on the amino acid alignment of the NS1 gene. The best amino acid substitution model defined by IQ-TREE was the Q.yeast + F + G4. *Tetraparvovirus* species are identified by color. The red color highlights the new sequence, while the white color represents unclassified viruses and the black color species from other genera.

Sequencing produced 43,168,902 reads, 468 of which were related to the Parvoviridae family. From them, a 5,258 nt contig was assembled with a genome read coverage of 11.1× and 54.8% of GC content. The putative NS1 gene extends from nucleotides 206 to 2,194, and the putative VP capsid gene from nucleotides 2,222 to 4,963. The genome showed the highest nucleotide identity with the *Tetraparvovirus didelphimorph1* (MG745671), which was 73.97%. It was also the closest in amino acid identity of the NS1 gene (80.96%), below the species demarcation criteria threshold. Based on this gene, the sequence grouped within the *Tetraparvovirus* monophyletic clade in a subclade with the opossum sequence (Fig. 1), which is from São Paulo State, Brazil (9). These findings suggest that the obtained coding-complete genome is very likely a new *Tetraparvovirus* species.

## Data Availability

The virus sequence and its host mtDNA sequence are the first version, PV693370.1 and PV684749.1, respectively. The raw sequencing data has the following SRA number: SRR33632182.

## References

[1] Cotmore SF, Agbandje-McKenna M, Canuti M, Chiorini JA, Eis-Hubinger A-M, Hughes J, Mietzsch M, Modha S, Ogliastro M, Pénzes JJ, Pintel DJ, Qiu J, Soderlund-Venermo M, Tattersall P, Tijssen P, ICTV Report Consortium. 2019. ICTV virus taxonomy profile: Parvoviridae. J Gen Virol 100:367–368. doi:10.1099/jgv.0.001212 30672729 PMC6537627

[2] Canuti M, Eis-Huebinger AM, Deijs M, de Vries M, Drexler JF, Oppong SK, Müller MA, Klose SM, Wellinghausen N, Cottontail VM, Kalko EKV, Drosten C, van der Hoek L. 2011. Two novel parvoviruses in frugivorous New and Old World bats. PLoS ONE 6:e29140. doi:10.1371/journal.pone.0029140 22216187 PMC3246463

[3] Chen S, Zhou Y, Chen Y, Gu J. 2018. Fastp: an ultra-fast all-in-one FASTQ preprocessor. Bioinformatics 34:i884–i890. doi:10.1093/bioinformatics/bty560 30423086 PMC6129281

[4] Kopylova E, Noé L, Touzet H. 2012. SortMeRNA: fast and accurate filtering of ribosomal RNAs in metatranscriptomic data. Bioinformatics 28:3211–3217. doi:10.1093/bioinformatics/bts611 23071270

[5] Li D, Liu CM, Luo R, Sadakane K, Lam TW. 2015. MEGAHIT: an ultra-fast single-node solution for large and complex metagenomics assembly via succinct de Bruijn graph. Bioinformatics 31:1674–1676. doi:10.1093/bioinformatics/btv033 25609793

[6] Buchfink B, Xie C, Huson DH. 2015. Fast and sensitive protein alignment using DIAMOND. Nat Methods 12:59–60. doi:10.1038/nmeth.3176 25402007

[7] Kearse M, Moir R, Wilson A, Stones-Havas S, Cheung M, Sturrock S, Buxton S, Cooper A, Markowitz S, Duran C, Thierer T, Ashton B, Meintjes P, Drummond A. 2012. Geneious Basic: an integrated and extendable desktop software platform for the organization and analysis of sequence data. Bioinformatics 28:1647–1649. doi:10.1093/bioinformatics/bts199 22543367 PMC3371832

[8] Minh BQ, Schmidt HA, Chernomor O, Schrempf D, Woodhams MD, von Haeseler A, Lanfear R. 2020. IQ-TREE 2: new models and efficient methods for phylogenetic inference in the genomic era. Mol Biol Evol 37:1530–1534. doi:10.1093/molbev/msaa015 32011700 PMC7182206

[9] de Souza WM, Dennis T, Fumagalli MJ, Araujo J, Sabino-Santos G, Maia FGM, Acrani GO, Carrasco A de O, Romeiro MF, Modha S, Vieira LC, Ometto T, Queiroz LH, Durigon EL, Nunes MRT, Figueiredo LTM, Gifford RJ. 2018. Novel parvoviruses from wild and domestic animals in Brazil provide new insights into parvovirus distribution and diversity. Viruses 10:143. doi:10.3390/v10040143 29565808 PMC5923437

